# Exploration of Sex and Age-Based Associations in Clinical Characteristics, Predictors of Severity, and Duration of Stay among COVID-19 Patients at the University Hospital of Saudi Arabia

**DOI:** 10.3390/healthcare11050751

**Published:** 2023-03-03

**Authors:** Rasha Assad Assiri, Asmatanzeem Bepari, Waseemoddin Patel, Syed Arif Hussain, Shaik Kalimulla Niazi, Asma Alshangiti, Safia Ali Alshangiti, Mary Anne Wong Cordero, Shazima Sheereen

**Affiliations:** 1 Department of Basic Health Sciences, College of Medicine, Princess Nourah bint Abdulrahman University, Riyadh 11671, Saudi Arabia; 2 Department of Pediatrics, King Abdullah bin Abdulaziz University Hospital, Riyadh 11564, Saudi Arabia; 3 Respiratory Care Department, College of Applied Sciences, Almaarefa University, Dariyah, Riyadh 13713, Saudi Arabia; 4 Department of Internal Medicine, King Abdullah bin Abdulaziz University Hospital, Riyadh 11564, Saudi Arabia; 5 Department of Preparatory Health Sciences, Riyadh Elm University, Riyadh 12611, Saudi Arabia; 6 College of Medicine, Princess Nourah bint Abdulrahman University, Riyadh 11671, Saudi Arabia; 7 Department of Pathology, Manipal Academy of Higher Education, Mangalore 576104, India

**Keywords:** COVID-19, clinical features, severity, age group, co-morbidities, Saudi Arabia, hospital stay, health systems

## Abstract

COVID-19 infection has a spectrum of variable clinical severity between populations because of their characteristic demographic features, co-morbidities, and immune system reactions. This pandemic tested the healthcare system’s preparedness, which depends on predictors of severity and factors related to the duration of hospital stays. Therefore, we carried out a single-center, retrospective cohort study in a tertiary academic hospital to investigate these clinical features and predictors of severe disease and study the different factors that affect hospital stay. We utilized medical records from March 2020 to July 2021, which included 443 confirmed (positive RT-PCR) cases. The data were explained using descriptive statistics and analyzed via multivariate models. Among the patients, 65.4% were female and 34.5% were male, with a mean age of 45.7 years (SD ± 17.2). We presented seven age groups with ranges of 10 years and noticed that patients aged 30–39 years old comprised 23.02% of the records, while patients aged 70 and above comprised 10%. Nearly 47% were diagnosed as having mild, 25% as moderate, 18% as asymptomatic, and 11% as having a severe case of COVID-19 disease. Diabetes was the most common co-morbidity factor in 27.6% of patients, followed by hypertension (26.4%). Our population’s predictors of severity included pneumonia, identified on a chest X-ray, and co-morbid conditions such as cardiovascular disease, stroke, ICU stay, and mechanical ventilation. The median length of hospital stay was six days. It was significantly longer in patients with a severe disease and who were administered systemic intravenous steroids. An empirical assessment of various clinical parameters could assist in effectively measuring the disease progression and follow-up with patients.

## 1. Introduction

Coronavirus disease 2019 (COVID-19), defined by the World Health Organization (WHO), is a highly transmittable acute respiratory disease caused by a novel strain of coronavirus, severe acute respiratory syndrome coronavirus 2 (nCoV-2019 or SARS-CoV-2), which belongs to the Coronaviridae family and caused the recent viral outbreak [1]. Its first case was identified in Wuhan, China, in December 2019, and within a short period of time, it had crossed most borders [2]. It spread to more than 200 countries, resulting in the first-ever pandemic caused by a coronavirus. As a result, WHO announced the outbreak of COVID-19 to be a “public health emergency of international consideration” on January 30 2020 [3]. In Saudi Arabia, the largest country in the Arabian Peninsula, the first confirmed case of COVID-19 was documented on March 2 2020 [4]. Saudi Arabia has a well-established healthcare system with 497 hospitals (287 are MOH hospitals), 99,617 total physicians, and 22.6 beds per 10,000 people. Among the MOH hospitals, 65 are accredited by the Central Board of Accreditation for Healthcare Institutions (CBAHI), established by the Ministry of Health [5]. In addition, several private and public hospitals have international accreditations, such as the Joint Commission International (JCI), which is accredited by the International Society for Quality in Health Care (ISQua), which serves as a safety collaborating center designated by the World Health Organization [6]. Nearly 75 Saudi hospitals are JCI-accredited [7].

Saudi authorities prepared public and private institutions to deal with the pandemic with strategic preparedness and a COVID-19 response plan, in line with the WHO operational planning guidelines to support the country. The authorities launched a governance system constituted of responsible committees to continuously monitor national and international updates, trace contacts, screen the population, increase awareness, and take appropriate actions to arrest the spread of this disease. Restrictions on social and religious gatherings, travel, and businesses were set ahead of the first 100 confirmed COVID-19 cases [8]. Mass screening programs were carried out in three stages. The first stage focused on screening individuals in highly populated districts through field tests in 807 locations; the second stage was facilitated through the Mawid app self-assessment tool, which classifies users as low- or high-risk. The low-risk group was the targeted population and was screened in designated primary care centers. The third stage was screening suspected COVID-19 cases with no symptoms at specialized drive-through testing centers, so-called takkad centers. The Hajj pilgrimage for 2020 was scaled down to confine participants, and no cases of COVID-19 were detected among pilgrims. As of 30 March 2020, the Saudi health authority announced that COVID-19 treatment is free for all citizens and residents. The country preserved all primary health services and immunization programs and supported all COVID-19 drugs and vaccine proposals [9]. According to the WHO, as of November 1, 2022, globally, over 600 million confirmed cases of COVID-19, including more than six million deaths, were reported; Saudi Arabia accounted for less than a million cases and around nine thousand deaths [10].

Usually, coronavirus causes a mild flu-like infection. However, like the severe acute respiratory syndrome (SARS) and Middle East respiratory syndrome (MERS), which caused severe outbreaks worldwide in the last decade, COVID-19 causes a spectrum of clinical features ranging from asymptomatic cases and mild upper respiratory infections to life-threatening pneumonia [11]. COVID-19 usually presents as a fever, cough, fatigue, and shortness of breath. Less frequent manifestations include a sore throat, chest pain, headache, diarrhea, and loss of taste or smell. Severe pneumonia can cause acute respiratory distress syndrome (ARDS), demanding respiratory support and extended care in intensive care, which can lead to life-threatening complications, multiple organ dysfunction syndrome, and death [11,12,13]. Despite the reports showing that SARS-CoV-2 centrally manifested as a respiratory infection, new data demonstrated that it must be considered a systemic disease infecting multiple systems, such as the gastrointestinal, immune, hematopoietic, cardiovascular, and respiratory systems [14].

Contemporary studies have documented that the clinical characteristics of the disease are variable between populations because of their distinctive demographic characteristics and co-morbidities, specifically correlating with the severe form of the disease compared to the milder form [15,16]. However, few studies have concentrated on discrepancies between sex and age clusters for clinical characteristics and severity among COVID-19 patients for an extended period in the early infection waves in Saudi Arabia. Therefore, recognizing these variations is often helpful in identifying the progression to a severe form of the disease and possibly optimizing COVID-19 case management [11,17,18,19]. In addition, this awareness helps in formulating preventive measures, recognizing the effect of COVID-19 on hospital capacity, and improving patient bed capacity and health systems through risk stratification.

King Abdullah bin Abdulaziz University Hospital (KAAUH), located in the capital city of Saudi Arabia, Riyadh—the region with the highest population—and one of the mainstream hospitals of this region, is a COVID-19 testing and vaccination center. It is on the Princess Nourah bint Abdulrahman University (PNU) campus, which has 20 colleges and 121 academic programs. It is a 400-bed, designated academic MOH hospital accredited by CBAHI and JCI; it is, therefore, one of the few hospitals that have national and international accreditations reflecting the commitment to the maintenance of high-quality health services and a compelling center for research documentation. Nearly 450 COVID-19 cases from March 2020 to July 2021 were treated at this hospital. During this period, the country faced its highest peak of cases, and most citizens and residents were not vaccinated. Therefore, we aimed to conduct a retrospective cohort study, investigating the differences in clinical variables and analyzing predictors of severity and factors that influence the duration of hospital stay among COVID-19-admitted cases. As far as our knowledge, to date, very few studies have targeted a more extended period for recruiting admitted COVID-19 patients (one and a half years) that focused on diverse age groups and sex-based differences for clinical characteristics, co-morbidities, severity, and duration of stay in Saudi Arabia.

### 1.1. Objectives of the Study

#### 1.1.1. Aim of the Study

The study’s goal was to evaluate the severity of diverse symptoms and signs that developed in patients with COVID-19 disease and analyze variations in different clinical factors with age clusters and sex in confirmed COVID-19 patients admitted to a university hospital in Saudi Arabia retrospectively.

#### 1.1.2. Specific Objectives

1. To assess the severity of different symptoms and signs of COVID-19 disease that developed in patients.
2. To investigate the differences in clinical variables with age, sex, and different co-morbidities.
3. To compare the outcome of COVID-19 patients by severity levels.
4. To assess the predictors of severity and duration of hospital stay (DoHS).

## 2. Materials and Methods

### 2.1. Study Design and Setting

This study was conducted as per the guidelines from STROBE (strengthening the reporting of observational studies in epidemiology) [20]. This was a single-center, non-interventional, non-exhaustive, retrospective cohort study, conducted at King Abdullah bin Abdulaziz University Hospital (KAAUH), Riyadh, Riyadh Province, Saudi Arabia. KAAUH is a referral hospital with nearly 400 beds and one of the designated COVID-19 hospitals and COVID-19 vaccination centers in Saudi Arabia (KSA).

### 2.2. Population

We included all patients with confirmed COVID-19 infection who were admitted to King Abdullah bin Abdulaziz University Hospital (KAAUH) between March 2020 and July 2021. Confirmed case of COVID-19 was defined as positive for SARS-CoV-2 virus through real-time reverse transcriptase–polymerase chain reaction (RT-PCR) assay on nasopharyngeal swab specimens. On admission, RT-PCR nasopharyngeal swabs were sent to all patients with clinical suspicion of COVID-19. The result was reported as either positive or negative and was available within 24 h. Therefore, we included 443 cases with positive RT-PCR results; cases with negative RT-PCR and incomplete clinical data were excluded.

### 2.3. Institutional Ethical Approval

The study was approved by Institutional Ethical and Review Board (PNU IRB and KAAUH IRBN), Riyadh, Saudi Arabia (PNU IRB number 21-0352, dated 16 September 2021, KAAUH IRB number RO2021-P-019, dated 3 October 2021).

### 2.4. Data Collection

A convenient sampling method for recruiting records from COVID-19 patients was used. Health Information Management (HIM) office provided Medical Record Numbers (MRN) of these confirmed COVID-19 patients admitted to Internal Medicine, Pulmonology, and Critical Care departments. Electronic medical record data were obtained using institutional software (TrakCare) and entered into the data collection form for detailed review. Data collected included: demographic characteristics (age, sex); co-morbidities, such as diabetes, hypertension, obesity, asthma, chronic obstructive pulmonary disease (COPD), chronic kidney disease (CKD), cardiovascular disease, thyroid disease, obesity, and immunosuppression; relevant clinical features (fever, cough, breathlessness, chest pain, loss of taste or smell, headache, myalgia, loose stools, respiratory rate, oxygen saturation (SpO2), chest X-ray); severity; and outcome (discharge or death), as displayed in Table 1.

### 2.5. Data Management and Analysis Plan

Data were analyzed using the JMP SAS statistical software. They were described through descriptive statistics, such as frequency, percentages, measures of central tendency, and proportions, and were analyzed via inferential statistical tests, such as chi-square test to assess categorical variables and multivariate models. Odds ratios (ORs) and their corresponding 95% CIs were calculated, and logistic regression analysis was applied to adjust for confounders of the association between different variables and case fatality of COVID-19. The values were compared at 0.05 level of significance to test the results of the study for the corresponding degrees of freedom.

## 3. Results

### 3.1. Age and Sex

**Figure 1:**
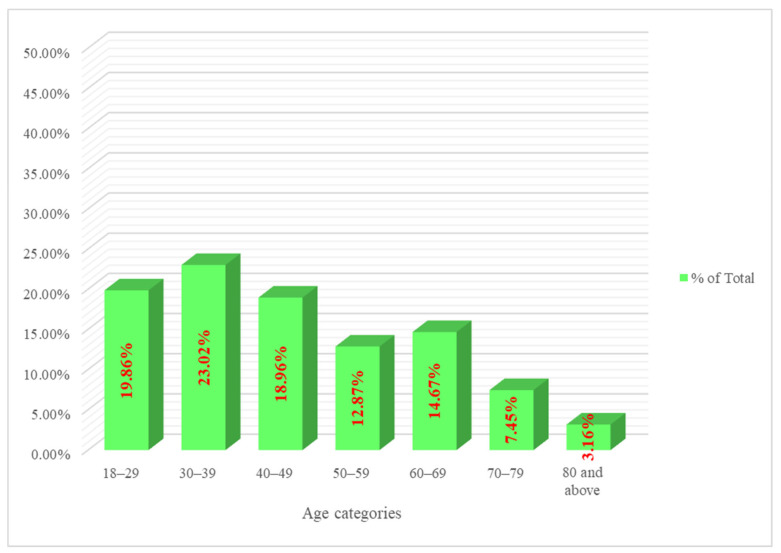
Age-wise distribution of patients of the cohort.

A total of 443 patients were included in the analysis; 65.4% of them were female and 34.5% were male, with a mean age of 45.7 years (SD ± 17.2). Of these, we presented seven age groups with ranges of 10 years and noticed that patients aged 30–39 years comprised 23.02% of the total, followed by patients aged 18–29 years (19.86%), 40–49 years (18.96%), 60–69 years (14.67%), and 50–59 years (12.87%), as depicted in Figure 1. Nearly 7.5 % of patients were in the range of 70–79 years and 3.2% were 80 and above. This lower number of patients aged 70 and above reflects the country’s expected life expectancy at birth of 75 years.

### 3.2. Clinical Characteristics of COVID-19 among Study Participants

Table 2 shows the frequency distribution of a variety of symptoms seen in our study participants on admission. The most common clinical manifestation was a fever (55.76%), followed by a cough (53.27%), dyspnea or shortness of breath (37.92%), headache (22.35%), diarrhea (20.32%), and myalgia (19.41%). A further analysis was conducted among age clusters and sex-based differences.

### 3.3. A. Different Age Groups

Table 3 displays the spectrum of clinical characteristics among the seven age clusters of the cohort. Fever, cough, and dyspnea were found to have a higher incidence in the age groups 60–69, 50–59, and 40–49 years and were statistically significant. Moreover, nearly 65% of “70–79 years” and “80 and above” patients reported these symptoms. Ageusia and abdominal pain rates were found to be comparatively higher in younger age groups, and were statistically significant; however, they remained low (around 2%), as seen in Table 3.

### 3.4. B. Sex-Based Differences

A higher percentage of males complained of a fever (*p* = 0.0103), a cough, dyspnea, ageusia, and arthralgia, while headache, myalgia, abdominal pain, and anosmia were seen comparatively more in females, but were not statistically significant except for fever, as displayed in Table 3.

### 3.5. Co-Morbidities in the Study Participants

**Figure 2:**
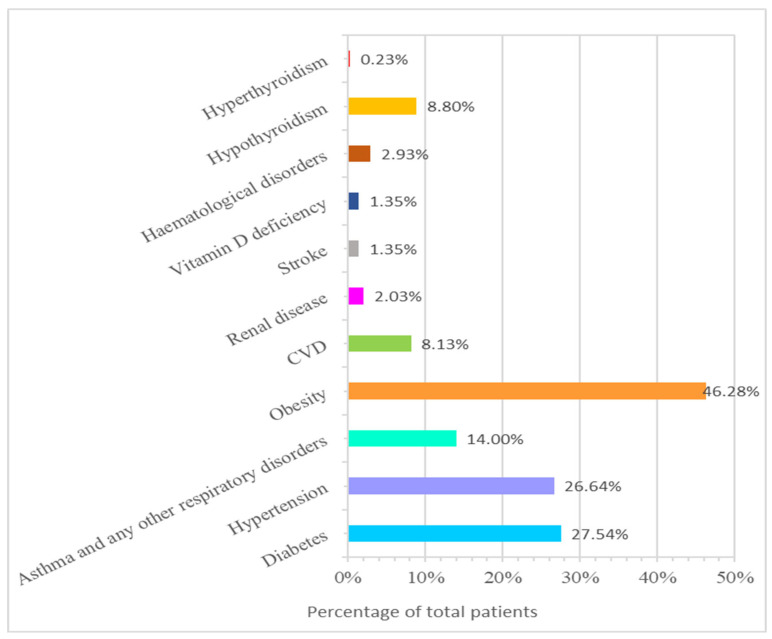
Descriptive statistics of different co-morbidities in the cohort.

In our study, 46.28% of patients were obese. Diabetes was the most common co-morbidity found in 27.6% of patients, followed by hypertension (26.4%) and asthma or other respiratory disorders (14%), as depicted in Figure 2.

### 3.6. A. Different Age Groups

**Figure 3:**
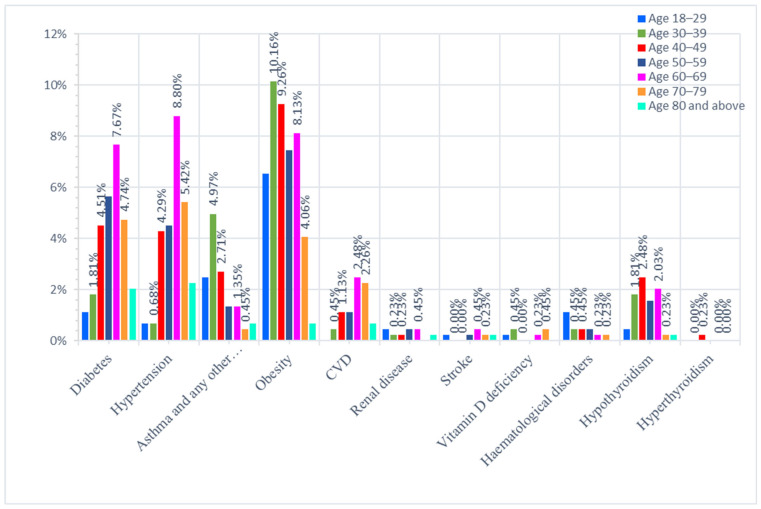
Age-wise distribution of co-morbidities in COVID-19 admitted patients.

Of the patients that were obese, nearly 10% belonged to the 30–39 years group, followed by the 40–49 years group, which comprised 9% of them. Hypertension (8.8%), diabetes (7.67%), and cardiovascular diseases, such as congestive heart failure and hypothyroidism (*p* = 0.0481 *), were higher in incidence among the age group 60–69 years and were statistically significant (*p* < 0.0001 ****). It was also seen that diabetes and HTN were notably more common in ages 70–79 years (4.74%, 5.42%), 50–59 years (5.65%, 4.5%), and 40–49 years (4.3%, 4.5%). While asthma and other respiratory disorders were comparatively higher in the younger age group of 30–39 years, as seen in Figure 3 and Table 4. A nominal logistic fit analysis was used to adjust for covariates of age and sex.

### 3.7. B. Sex-Based Differences

A higher percentage of males suffered HTN (*p* = 0.0119) and cardiovascular disease (*p* = 0.0467 *), while obesity (0.0467 *), hypothyroidism (0.0448 *), and a vitamin D deficiency (0.0235 *) had comparatively higher rates in females and were statistically significant, as displayed in Table 4.

### 3.8. The Severity of COVID-19

**Figure 4:**
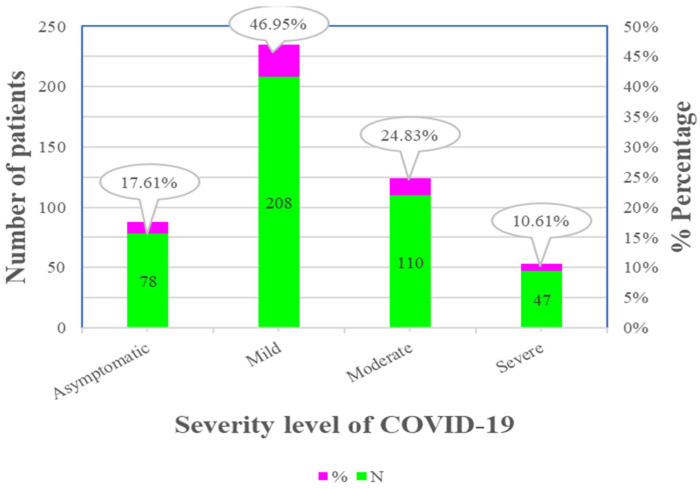
Descriptive statistics of severity of COVID-19 among study participants.

Nearly 47% of patients were diagnosed as having mild, 25% as moderate, 18% as asymptomatic, and 11% as having severe COVID-19 disease, as depicted in Figure 4. Pneumonia on chest X-rays was seen in 48% of patients, while 18.08% required oxygen support on arrival, 14.22% were transferred to the ICU, and 4.07% needed intubation. There was a statistically significant difference between different age groups and sexes, as seen in Table 5. A higher percentage of elderly patients needed oxygen support on arrival, were admitted to the ICU, and required intubation.

For further analysis, the four severity groups were narrowed down into two groups, ‘mild disease’ with asymptomatic–mild disease patients together, and ‘severe disease’ comprising moderate–severe disease. Table 6A–C represent the association found among various clinical parameters: age, sex, and interventions opted by the severity of the disease.

### 3.9. Predictors of Severity and Duration of Hospital Stay (DoHS)

A multivariate logistic regression analysis indicated that the patient’s respiratory need for O_2_ on arrival, the presence of pneumonia on chest X-ray, admission to the ICU, and existing cardiovascular disease (MI, CHF, IHD) and stroke were significant predictors for the severity (Table 7).

**Figure 5:**
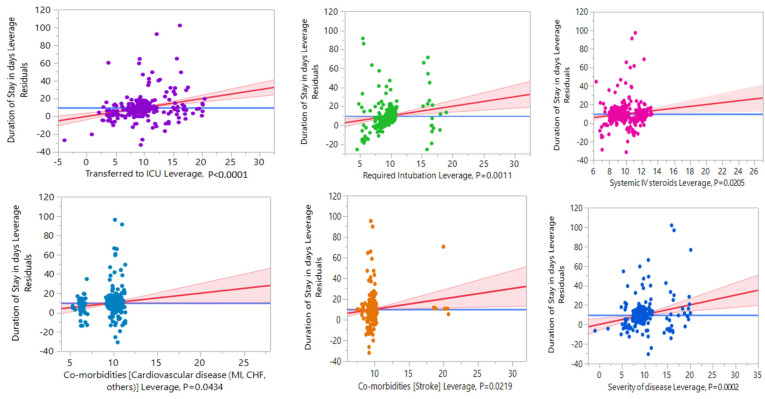
Leverage plots of independent predictors with duration of stay in hospital.

Figure 5 displays the leverage plots of independent predictors with the duration of stay in the hospital. Table 8 shows the duration of hospital stay (DoHS) in days for the patients, with a maximum of 118, an average of 9.72 ± 14.2 (SD), and a median of 6 days. A comparison of the factors showed that patients with severe symptoms were associated with a more prolonged DoHS than those with mild-to-moderate symptoms (mean 32 and 5.5 days, estimate = 3.29, *p* < 0.0018, respectively). The results also indicated that patients admitted or transferred to the ICU were associated with a significantly longer DoHS (mean 29.26 and 6.4 days, estimate = 6.41, *p* < 0.0001) than patients in the general isolation ward. Similarly, patients on mechanical ventilation, CVD, or stroke, were associated with a longer DoHS than those without these disorders (estimate = 5.65, *p* = 0.0011; 2.19, *p* = 0.04; and 5.6 days, *p* = 0.02, respectively). We also discovered that patients who received systemic intravenous steroids had a longer DoHS (estimate = 1.75, *p* = 0.02).

### 3.10. The Outcome of COVID-19 Patients with Severity Level, Age, and Sex

**Figure 6:**
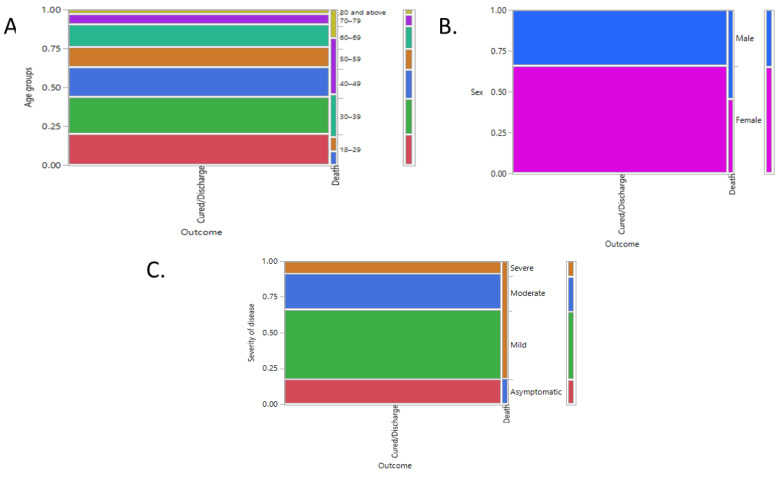
Mosaic plot displaying contingency analysis of outcome by (**A**) age groups, (**B**) sex, and (**C**) severity of disease.

Nearly 97.5% of the patients were cured and discharged, while 2.5% succumbed to the disease. As displayed in Table 9 and Figure 6, this 2.5% comprised elderly males diagnosed with severe disease on admission.

## 4. Discussion

In our study, we explained the different clinical factors, co-morbidities, and severity of 443 documented patients with COVID-19 at KAAUH, a JCIA- and CBAHI-accredited MOH hospital in Riyadh, Saudi Arabia, along with predictors of severity and characteristics associated with more extended hospital stays. Hospital accreditation generates a positive impact on most patient safety indicators and thereby is one of the driving forces towards improving quality healthcare in KSA. The Riyadh region is the most COVID-19-infected province of Saudi Arabia, according to the MOH-launched “COVID-19 Statistics E-Platform” [21]. One of the limitations of the previous studies is the main focus on midlife adults and older adults aged 60 and above. However, further subgroup analysis of elderly patients is present in very few studies; thereby, exploring the age-related clinical features of COVID-19 across all age groups is a matter of interest for study. Another typical limitation in earlier research works was the insufficient or missed information about the healthcare accreditation standards of the hospitals studied. In our study, the early midlife age range of 30–39 years comprised one-fourth of our patients, followed by younger patients who comprised one-fifth (19.86%), while the remaining one-fourth were 40–49 years (18.96%) and 50–59 years (12.87%), as depicted in Figure 1. The previous reports showed similar results: the middle-aged “40 to 60 years” were the most commonly infected group [22,23,24]. While only one-tenth of patients were in the ranges of 70–79 years and 80 and above. However, individuals of all age groups can be infected by the virus [23,25]. Nearly two-thirds of the patients were female (65.4%) and the remaining one-third (35%) were male, with a mean age of 45.7 years (SD, ±17.2). However, a single-arm meta-analysis indicated that men represent a significantly higher percentage of COVID-19 patients at 60% (95% CI [0.54, 0.65]) [26], and other previous studies have also reported a significantly higher number of men having the infection compared to women [22,27]. In addition, previous studies have recorded more men than women infected with other coronavirus infections, such as SARS-CoV [28] and MERS-CoV [29]. The disproportionate number of female hospitalizations in the present study can be due to the greater input of female patients from the affiliated women’s university (PNU).

Our findings indicated that a fever (55.76%), a cough (53.27%), and dyspnea (shortness of breath, 37.92%) were the most frequent symptoms in COVID-19 patients, which is in accordance with multiple earlier studies [30,31,32,33]. These symptoms were more commonly found in older patients of 60–69 years old, 70–79 years old, or 80 years old and above, which were statistically significant compared to younger groups (Figure 2 and Table 3). Nearly one-fourth of patients complained of headache (22.35%), diarrhea (20.32%), or myalgia (19.41%). It was noted that ageusia and abdominal pain were statistically higher in younger age groups than in older patients; however, their incidence was low (around 2%). Research evidence indicates that the disorder’s complete clinical characteristics are nonspecific and unclear, as the associated manifestations range from mild to severe, and resulting in death in several patients [10,34]. There were no differences between the sexes for most symptoms among the COVID-19 patients, except for the fever, which was higher in males than females (*p* = 0.0103, Table 3). Previous studies have consistently noted poorer outcomes in men in terms of morbidity and mortality, confirming the male sex as an independent risk aspect for COVID-19 [35,36,37]. The more robust innate and adaptive immune response in females can be attributed to numerous factors, though primarily to estrogen being immune strengthening as opposed to testosterone being immune suppressing [38].

It was found that diabetes was the most common co-morbidity (27.6%) seen in the admitted COVID-19 patients of our study, followed by hypertension (26.4%), which is comparable to the prevalence in the general population. Our findings revealed that, among different age clusters, many 60–69-years-old patients infected by SARS-CoV-2 had chronic underlying diseases, including hypertension (8.8%), diabetes (7.67%), and cardiovascular diseases such as congestive heart failure (*p* < 0.0001 ****) and hypothyroidism (*p* = 0.0481 *), which were statistically noteworthy, as depicted in Table 4 and Figure 3. It was also seen that diabetes and HTN were notably more common in ages “70–79” years (4.74%, 5.42%), 50–59 years (5.65%, 4.5%), and 40–49 years (4.3%, 4.5%). This agrees with an earlier report in which COVID-19 patients were documented to have co-morbid disorders [22,39]. In addition, research data show that patients aged 60 years old and above are at a higher risk than children, who are less likely to have an infection or show mild to asymptomatic infections [40]. In addition, our study revealed that 60–70% of patients in the “70–79 years” and “80 and above” ranges were suffering from diabetes or hypertension, and nearly 55% of each group were obese. The augmented risk of acquiring severe COVID-19 complications in older people with co-morbid conditions such as diabetes or hypertension has been well documented in the literature [22]. Previous studies from the United States, Italy, and China have noted that the diabetic population is at a more prominent risk for disease complications and infection susceptibility [41]. The most extensive study of COVID-19 cases (72,314 cases) from China showed a higher incidence of mortality among patients with diabetes and COVID-19 (2.3% without diabetes vs. 7.3% with the disease) [42]. In contrast, the occurrence of asthma and other respiratory disorders was comparatively higher in the younger age group of 30–39 years (Figure 3 and Table 4). Therefore, identifying host risk characteristics associated with severe COVID-19 infections may allow the design of specific approaches to prevent and treat the disease [39].

There were differences among the sexes in co-morbidities among our cohort of admitted COVID-19-positive patients. It was seen that a higher percentage of males suffered HTN (*p* = 0.0119 *) and cardiovascular disease (*p* = 0.0467 *), while obesity (0.0467 *), hypothyroidism (0.0448 *), and vitamin D deficiencies (0.0235 *) were comparatively higher in females and were statistically significant, as displayed in Table 4. The lower vitamin D level has been invariably associated with an augmented risk of upper respiratory tract infections and pneumonia, secondary to weak immune systems and raised inflammatory cytokines [43]. Research studies also link vitamin D depletion to poor COVID-19 prognosis and mortality [44,45].

We found that 47% of the patients were diagnosed as having mild, 25% as moderate, 18% as asymptomatic, and 11% as having severe COVID-19, as depicted in Figure 5. Nearly 15% of the patients were transferred to the ICU, which is sensitive to understanding the severity of the disease, wherein 50% of them (7% of the total, *p* < 0.0001 ****) were elderly patients, followed by the 40–49 years age group (3.4% of the total), which was found to be statistically significant compared to the younger age group, as displayed in Table 5. Additionally, more males (18.95%, *p* = 0.0417 *) were admitted to the ICU. Similar findings were reported in a systematic review and meta-analysis comprising seven studies (1813 COVID-19 cases with a more significant proportion of male patients), demonstrating that patients admitted to the ICU were older (mean age = 62.4 years) compared with non-ICU patients (mean age = 46 years). Additionally, they found that 1591 primarily older male patients with co-morbid diseases admitted to ICUs had moderate to severe ARDS [46,47]. In our study, pneumonia discovered on chest X-rays was seen in 48% of patients, 18.08% required oxygen support on arrival, and 4.07% required intubation. In our study, oxygen on arrival was required more in the age group of 60–69 years, followed by 40–49 years, 50–59 years, and 70–79 years (*p* < 0.0001 ****), and was managed with systemic steroids (11.29%, *p* < 0.0001 ****). Our study revealed that more patients aged 70–79 years and 60–69 years required intubation, which was statistically significant compared to younger patients (*p* = 0.002). Therefore, the exploration of age-related effects on COVID-19 severity helps us progress strategies required for developing the healthcare system in COVID-19-treating hospitals. In addition, more males (18.95%, *p* = 0.0417 *) were admitted to ICU, and there was a statistical difference between the sexes. A higher percentage of males were given oxygen support on arrival (*p* = 0.0081 *) and treated with systemic steroids (*p* = 0.0294 *), while no difference in sexes was found for intubation. Similar findings were reported by Chen et al., who proposed that nCoV-2019 is more likely to infect elderly adult men with chronic co-morbidities due to these patients’ more vulnerable immune functions [22].

Furthermore, the analysis of two groups of mild and severe COVID-19 disease showed that a fever, a cough, and dyspnea were more common in severe disease cases. At the same time, diarrhea and ageusia were more common in mild disease cases (Table 6A–C). We used a multiple logistic regression and found that pneumonia discovered by chest X-rays and co-morbid conditions, such as cardiovascular disease, stroke, an ICU stay, and mechanical ventilation, were predictors of severity depicting statistically significant differences (Table 7). Guan et al. documented that patients with severe COVID-19 disease were older than those with non-severe disease, and any co-morbidity was more expected among patients with severe disease than those with non-severe COVID-19 disease [48]. Another study documented similar findings, wherein it was seen that older age and hypertension were independently associated with severe disease at admission [39].

The median period of hospital stay in our study was six days. A similar result was reported by Alwafi et al. (range: 0–55 days) [49]. Alghamdi et al. presented that the DoHS in Saudi Arabia among COVID-19 patients ranged from 4 to 15.6 days [50]. We found that it was significantly longer in patients with severe disease who needed oxygen support or mechanical ventilation, as expected, and also that a longer stay occurred in patients with CVD or stroke who were administered systemic intravenous steroids, as depicted in Figure 6 and Table 8. Wang et al. reported similar results, showing that clinical severity was firmly correlated to the period of stay (*p* < 0.01) and that a longer DoHS was associated with patients with admission to a provincial hospital, 45 years of age or older, and severe illness [51].

Our analysis’ mortality rate for COVID-19 was 2.5%, proximate to the 3.4% reported in the literature [30]. Previous studies found that the prevailing fatality rate of the disease was in the range of 3% to 14% [52]. In severe cases, the virus causes alveolar damage, leading to advanced respiratory failure, and causing death [34,53]. In our study, the mortality rate was relatively high among the elderly (*p* = 0.0006 *) (Figure 6, those admitted to the ICU (*p* < 0.0001 *), and patients with pneumonia on chest X-ray (*p* < 0.0001 ****), which resembles previous findings. Research evidence demonstrates that the aged and those with underlying chronic disorders produce severe and lethal respiratory failure because of alveolar injury from the virus [22,23,27]. In our study, the age groups of 70–79 years and 80 and above were significantly associated with in-hospital mortality. In addition, our study revealed that nearly 55.67% of patients aged “70–79 years” and the majority (78.5%) of patients aged “80 and above” were suffering from severe disease at the time of admission, and nearly 15% of each group succumbed to the disease. This was compared to another study conducted by Abolfoutoh et al., wherein mortality was high among patients aged 70 and above [54]. A meta-analysis, including studies from different countries—fifty from China, three from the USA, and one each from Germany, Iran, Italy, Singapore, South Korea, and the UK—revealed that patients aged 70 years and above have a higher infection threat, severity, and mortality risk compared with patients younger than 70 years [55].

Moreover, ICU patients were more likely to receive prolonged treatment and mechanical ventilation. No independent mortality association was observed in the present study with any co-morbidities [55]. However, established risk factors such as CVD, lung infiltrates, and stroke were noted to be substantial risk factors for severe COVID-19 in our study. It nonetheless suggests that the increased risk for worse outcomes is the accumulative effect of clustering with cardiometabolic multimorbidity or chronic diseases, which increases complications [56,57]. Most of these characteristics have been connected to advancing acute respiratory distress syndrome (ARDS), secondary to SARS-CoV and MERS-CoV [58]. All these risk factors, together with DM and HTN, lead to worsening pre-existing chronic inflammation, progressing to cytokine storm and prompt impairment of the endothelial function if left untreated [59]. Additionally, previous studies indicated that HTN, renal failure, and CVD raised the death risk in COVID-19 cases [34,60,61,62]. Furthermore, ACE inhibitors as antihypertensive are linked between hypertension and COVID-19 severity as ACE2 serves a role in SARS infections [63]. Table 10 summarizes the important highlights of the current study in comparison to the available literature.

In comparing the death rate of COVID-19 to that of other coronaviruses, the available data demonstrate that COVID-19 infection resulted in a lower mortality rate than that documented for SARS (9.60%) and MERS (34.4%) [60]. Thus, comprehensive analyses of the pathogenic and virulence mechanisms of SARS-CoV, SARS-CoV-2, and MERS-CoV are required to demonstrate these deviations.

We acknowledge some limitations. Our findings are confined to the accuracy of medical record-keeping, given its retrospective design. In addition, the single-centric nature of the investigation limits the generalization of results to the general population as the study sample is limited to 1 hospital among 497 hospitals (of which 65 are accredited by the Ministry of Health). Therefore, the study sample is not fully representative of COVID-19 patients admitted to hospitals in Saudi Arabia during the study period, which directs future research toward multicentric studies and huge sample sizes. Despite these limitations, the results of the present study are strong and add significance to the literature on COVID-19 patients within the Arab region, as it is a study performed in a hospital with international accreditation in the capital city of KSA, in a region with the highest COVID-19 infection rate, is longer than one and a half years in duration, thoroughly describes hospitalized patients during earlier waves, and differentiates clinical characteristics based on sex, seven different age clusters from “18–29 years” to “80 and above”, severity and its predictors, duration of hospital stay, and outcomes.

## 5. Conclusions

In summary, this study, conducted in a JCI- and CBAHI-accredited MOH hospital in Riyadh, Saudi Arabia, discovered that COVID-19 patients were most likely to present with a mild fever, cough, and shortness of breath on admission. There was an increased disease severity rate in elderly male patients aged “70 and above” who were transferred to the ICU, showed pneumonia on a chest X-ray, required intubation, and had a higher incidence of various co-morbidities, such as hypertension and cardiovascular diseases. In contrast, younger female patients suffered from vitamin D deficiency, obesity, and hypothyroidism. Moreover, the older age groups of 70–79 years and 80 years and above were significantly associated with in-hospital mortality. The hospital’s preparedness and quality of care were reflected in the lower mortality rate and the average length of hospital stay. More extensive epidemiologic studies and randomized trials covering multiple institutions are needed to determine a more accurate in-hospital severity and mortality rate in the country. Therefore, age and sex must be considered when estimating the clinical findings, severity, and mortality of COVID-19. This may improve the management of potentially severe COVID-19 patients by ensuring appropriate resource allocations and putting forward preventative measures.

## Figures and Tables

**Table 1:** Classification of severity of COVID-19.

Asymptomatic	Mild COVID-19	Moderate COVID-19	Severe COVID-19	
			Severe Cases	Critical Cases
COVID-19 PCR + ve, no symptoms	Symptoms (fever, cough, myalgia), RR < 24, SPo2 > 94% in room air, no pneumonia	Symptoms with shortness of breath, RR 24–30, SPo2 90–94 in room air, pneumonia	Pneumonia plus any one of the following: RR > 30, SPo2 < 90 in room air, severe respiratory distress, requiring respiratory support	ARDS, respiratory failure requiring ventilation support, sepsis, septic shock, MODS

**Table 2:** Clinical characteristics displayed by the study participants.

Clinical Characteristics	N	Percentage
Fever	247	55.76%
Cough	236	53.27%
Dyspnea	168	37.92%
Headache	99	22.35%
Nausea/Vomiting	76	17.16%
Myalgia	86	19.41%
Diarrhea	90	20.32%
Ageusia	28	6.32%
Abdominal pain	36	8.13%
Arthralgia	11	2.48%
Anosmia	15	3.39%

**Table 3:** Association between clinical symptoms with age clusters and sex (N = 443). *p* < 0.05 (*), *p* < 0.01 (**), *p* < 0.0001 (****).

Symptoms	Age Groups (Years)								Sex		
	18–29	30–39	40–49	50–59	60–69	70–79	80 and Above	*p*-Value	Female %	Male %	*p*-Value
Fever								<0.0001 ****			0.0103 *
Yes	8.80%	9.03%	11.51%	9.03%	10.38%	4.97%	2.03%		51.38%	64.05%	
No	11.06%	14.00%	7.45%	3.84%	4.29%	2.48%	1.13%		48.62%	35.95%	
Cough								<0.0001 ****			0.3679
Yes	8.35%	8.35%	10.84%	9.48%	9.48%	4.97%	1.81%		51.72%	56.21%	
No	11.51%	14.67%	8.13%	3.39%	5.19%	2.48%	1.35%		48.28%	43.79%	
Dyspnea								<0.0001 ****			0.4136
Yes	3.61%	6.09%	7.45%	7.45%	8.80%	3.16%	1.35%		36.55%	40.52%	
No	16.25%	16.93%	11.51%	5.42%	5.87%	4.29%	1.81%		63.45%	59.48%	
Headache								0.09			0.1329
Yes	6.09%	5.42%	3.39%	3.16%	3.39%	0.68%	0.23%		24.48%	18.30%	
No	13.77%	17.61%	15.58%	9.71%	11.29%	6.77%	2.93%		75.52%	81.70%	
Nausea/Vomiting								0.058			0.8424
Yes	3.61%	2.48%	3.84%	2.93%	3.61%	0.45%	0.23%		16.90%	17.65%	
No	16.25%	20.54%	15.12%	9.93%	11.06%	7.00%	2.93%		83.10%	82.35%	
Myalgia								0.438			0.2299
Yes	2.48%	4.06%	4.06%	3.39%	3.39%	1.58%	0.45%		21.03%	16.34%	
No	17.38%	18.96%	14.90%	9.48%	11.29%	5.87%	2.71%		78.97%	83.66%	
Diarrhoea								0.180			0.9834
Yes	4.06%	3.16%	5.64%	3.16%	2.71%	1.13%	0.45%		20.34%	20.26%	
No	15.80%	19.86%	13.32%	9.71%	11.96%	6.32%	2.71%		79.66%	79.74%	
Ageusia								0.006 **			0.3459
Yes	1.58%	2.26%	2.03%	0.23%	0.00%	0.23%	0.00%		5.52%	7.84%	
No	18.28%	20.77%	16.93%	12.64%	14.67%	7.22%	3.16%		94.48%	92.16%	
Abdominal pain								0.025 *			0.0937
Yes	2.48%	1.81%	2.03%	0.00%	1.13%	0.23%	0.45%		9.66%	5.23%	
No	17.38%	21.22%	16.93%	12.87%	13.54%	7.22%	2.71%		90.34%	94.77%	
Anosmia								0.212			0.5056
Yes	0.45%	1.58%	0.90%	0.23%	0.23%	0.00%	0.00%		3.79%	2.61%	
No	19.41%	21.44%	18.06%	12.64%	14.45%	7.45%	3.16%		96.21%	97.39%	
Arthralgia								0.550			0.1697
Yes	0.45%	0.90%	0.23%	0.23%	0.68%	0.00%	0.00%		1.72%	3.92%	
No	19.41%	22.12%	18.74%	12.64%	14.00%	7.45%	3.16%		98.28%	96.08%	

**Table 4:** Association between co-morbidities with age clusters and sex (N = 443). *p* < 0.05 (*), *p* < 0.0001 (****).

Co-Morbidities	Age Groups (Years)								Sex		
	18–29	30–39	40–49	50–59	60–69	70–79	80 and Above	*p*-Value	Female %	Male %	*p*-Value
Diabetes								<0.0001 ****			
Yes	1.13%	1.81%	4.51%	5.64%	7.67%	4.74%	2.03%		25.17%	32.03%	0.1272
No	18.74%	21.22%	14.45%	7.22%	7.00%	2.71%	1.13%		74.83%	67.97%	
Hypertension								<0.0001 ****			0.0119 *
Yes	0.68%	0.68%	4.29%	4.51%	8.80%	5.42%	2.26%		22.76%	33.99%	
No	19.19%	22.35%	14.67%	8.35%	5.87%	2.03%	0.90%		77.24%	66.01%	
Asthma and any other respiratory disorders								0.1663			0.3063
Yes	2.48%	4.97%	2.71%	1.35%	1.35%	0.45%	0.68%		12.76%	16.34%	
No	17.38%	18.06%	16.25%	11.51%	13.32%	7.00%	2.48%		87.24%	83.66%	
Obesity								0.102			0.0467 *
Yes	6.55%	10.16%	9.26%	7.45%	8.13%	4.06%	0.68%		52.76%	33.99%	
No	13.32%	12.87%	9.71%	5.42%	6.55%	3.39%	2.48%		47.24%	66.01%	
CVD								<0.0001 ****			0.0467 *
Yes	0.00%	0.45%	1.13%	1.13%	2.48%	2.26%	0.68%		6.21%	11.76%	
No	19.86%	22.57%	17.83%	11.74%	12.19%	5.19%	2.48%		93.79%	88.24%	
Renal disease								0.6263			0.1933
Yes	0.45%	0.23%	0.23%	0.45%	0.45%	0.00%	0.23%		1.38%	3.27%	
No	19.41%	22.80%	18.74%	12.42%	14.22%	7.45%	2.93%		98.62%	96.73%	
Stroke								0.2038			0.3252
Yes	0.23%	0.00%	0.00%	0.23%	0.45%	0.23%	0.23%		1.72%	0.65%	
No	19.64%	23.02%	18.96%	12.64%	14.22%	7.22%	2.93%		98.28%	99.35%	
Vitamin D deficiency								0.2782			0.0235 *
Yes	0.23%	0.45%	0.00%	0.00%	0.23%	0.45%	0.00%		2.07%	0.00%	
No	19.64%	22.57%	18.96%	12.87%	14.45%	7.00%	3.16%		97.93%	100.00%	
Hematological disorders								0.7098			0.3623
Yes	1.13%	0.45%	0.45%	0.45%	0.23%	0.23%	0.00%		3.45%	1.96%	
No	18.74%	22.57%	18.51%	12.42%	14.45%	7.22%	3.16%		96.55%	98.04%	
Hypothyroidism								0.0481 *			0.0448 *
Yes	0.45%	1.81%	2.48%	1.58%	2.03%	0.23%	0.23%		10.69%	5.23%	
No	19.41%	21.22%	16.48%	11.29%	12.64%	7.22%	2.93%		89.31%	94.77%	
Hyperthyroidism								0.7658			0.357
Yes	0.00%	0.00%	0.23%	0.00%	0.00%	0.00%	0.00%		0.34%	0.00%	
No	19.86%	23.02%	18.74%	12.87%	14.67%	7.45%	3.16%		99.66%	100.00%	

**Table 5:** Association between interventions by age groups and sexes (N = 443). *p* < 0.05 (*), *p* < 0.01 (**), *p* < 0.0001 (****).

Parameters	Age Groups (Years)								Sex		
	18–29	30–39	40–49	50–59	60–69	70–79	80 and Above	*p*-Value	Female %	Male %	*p*-Value
Required O_2_ on arrival								<0.0001 ****			0.0081 *
Yes	1.13%	1.35%	4.06%	2.71%	4.97%	2.48%	1.35%		14.48%	24.84%	
No	18.74%	21.67%	14.90%	10.16%	9.71%	4.97%	1.81%		85.52%	75.16%	
Systemic IV steroids								<0.0001 ****			0.0294 *
Yes	1.35%	3.39%	4.97%	4.74%	6.32%	3.16%	1.81%		22.41%	32.03%	
No	18.51%	19.64%	14.00%	8.13%	8.35%	4.29%	1.35%		77.59%	67.97%	
Transferred to ICU								<0.0001 ****			0.0417 *
Yes	0.90%	0.90%	3.39%	2.26%	3.61%	2.48%	0.68%		11.72%	18.95%	
No	18.96%	22.12%	15.58%	10.61%	11.06%	4.97%	2.48%		88.28%	81.05%	
Required intubation								0.0024 **			0.0609
Yes	0.00%	0.23%	0.90%	0.68%	1.13%	1.13%	0.00%		2.76%	6.58%	
No	19.91%	22.85%	17.87%	12.22%	13.57%	6.33%	3.17%		97.24%	93.42%	
Home isolation	2.26%	3.61%	3.16%	0.23%	0.68%	0.45%	0.00%	0.0037 **	11.38%	8.50%	0.3373

**Table 6:** A: Association analysis of clinical characteristics by COVID-19 severity (N = 443). *p* < 0.05 (*), *p* < 0.01 (**), *p* < 0.0001 (****); B: Association analysis of COVID-19 severity by age groups and sex (N = 443). *p* < 0.01 (**), *p* < 0.0001 (****); C: Association analysis of interventions and chest X-ray with the severity of the disease (N = 443). *p* < 0.05 (*), *p* < 0.0001 (****).

A			
Clinical Parameters	Severity of Disease		
	Mild %	Severe %	*p*-Value
I. Symptoms			
Fever	45.1	75.16	<0.0001 ****
Cough	41.26	75.16	<0.0001 ****
Dyspnea	24.83	61.78	<0.0001 ****
Headache	24.48	18.47	0.1424
Nausea/Vomiting	15.73	19.75	0.2879
Myalgia	17.83	22.29	0.2597
Diarrhea	17.13	26.11	0.0264 *
Ageusia	8.39	2.55	0.0096 **
Abdominal pain	9.09	6.37	0.3075
Arthralgia	3.15	1.27	0.2016
Anosmia	3.5	3.18	0.8616
II. Co-morbidities			
Diabetes	21.51	40.4	<0.0001 ****
Hypertension	45.22	54.78	<0.0001 ****
Asthma and any other RS disorders	12.54	16.56	0.2567
Obesity	44.8	50.33	0.2729
Cardiovascular disease (MI, CHF, others)	3.94	15.23	<0.0001 ****
Renal disease	2.15	1.99	0.9095
Cancer/leukemia	0.00%	0.00%	
Stroke	1.08	1.99	0.4523
Vitamin D deficiency	1.79	0.66	0.3114
Hematological disorders	3.58	0.66	0.067
Hypothyroidism	7.53	10.6	0.2851
Hyperthyroidism	0.36	0.00%	0.352
**B**			
	**Mild**	**Severe**	***p*** **-Value**
**I. Age Groups (Years)**			
18–29	27.27% (78)	6.37% (10)	<0.0001 ****
30–39	29.37% (84)	11.46% (18)	
40–49	16.78% (48)	22.93% (46)	
50–59	10.49% (30)	17.20% (27)	
60–69	9.79% (28)	23.57% (37)	
70–79	5.24% (15)	11.46% (18)	
80 and Above	1.05% (3)	7.01% (11)	
II. Gender			
Female	70.98%	55.41%	0.0011 **
Male	29.02%	44.59%	
**C**			
**Parameters**	**Severity of Disease**		
	**Mild %**	**Severe %**	***p*** **-Value**
Home isolation	12.59	6.37	0.0336 *
Required O_2_ on arrival	3.5	44.59	<0.0001 ****
Transferred to ICU	0.7	38.85	<0.0001 ****
Required intubation	0.35	10.9	<0.0001 ****
Chest X-ray: pneumonia	23.78	92.99	<0.0001 ****
Systemic IV steroids	11.89	50.96	<0.0001 ****
Systemic oral steroids	22.93	6.64	<0.0001 ****

**Table 7:** Multiple regression results of predictors of severe COVID-19 (N = 443). *p* < 0.05 (*), *p* < 0.01 (**), *p* < 0.001 (***), *p* < 0.0001 (****).

	Level1	Level2	Odds Ratio	Lower 95%	Upper 95%	Prob > Chisq
**Sex**	Male	Female	1.49	0.74	2.98	0.26
**Required O_2_ on arrival**	Yes	No	7.89	2.64	23.60	0.0002 ***
**Transferred to ICU**	Yes	No	26.01	3.52	192.32	0.0014 **
**Diabetes**	Yes	No	0.88	0.39	1.96	0.75
**Hypertension**	Yes	No	0.79	0.33	1.86	0.58
**Asthma and any other respiratory disorders**	Yes	No	2.40	0.95	6.06	0.06
**CVD (MI, CHF, others)**	Yes	No	3.37	1.04	10.89	0.0425 *
**Renal disease**	Yes	No	0.14	0.01	4.13	0.26
**Stroke**	Yes	No	20.52	1.29	326.53	0.0324 *
**Hematological disorders**	Yes	No	0.21	0.01	5.62	0.35
**Hypothyroidism**	Yes	No	1.15	0.37	3.56	0.80
**Hyperthyroidism**	Yes	No	0.00	0.00		1.00
**Chest X-ray: Pneumonia**	Yes	No	27.90	11.63	66.96	<0.0001 ****

**Table 8:** Independent factors associated with the length of hospital stay obtained using the standard least squares method. *p* < 0.05 (*), *p* < 0.01 (**), *p* < 0.001 (***); *p* < 0.0001 (****).

	Duration of Hospital Stay		Standard Least Squares Method Estimates			
Term	Mean	Std ERROR	Estimate	Std Error	t Ratio	Prob > |t|
Duration of hospital stay (days): maximum = 118, minimum = 1, mean = 9.72 ± 14.2 (SD), median = 6						
Intercept			24.493488	7.154958	3.42	0.0007 ***
Systemic IV steroids						
No	6.5915	7.1781791	−1.756811	0.755274	−2.33	0.0205 *
Yes	18.7281	7.2112043	Reference			
Transferred to ICU						
No	6.4316	7.2047820	−6.417144	1.196474	−5.36	<0.0001 ****
Yes	29.8871	7.3034964	Reference			
Required intubation						
No	8.3420	7.1128093	−5.656731	1.717231	−3.29	0.0011 **
Yes	42.2222	7.5955612	Reference			
Cardiovascular disease (MI, CHF, others)						
No	9.5074	7.0680036	2.1945388	1.082989	2.03	0.0434 *
Yes	12.1389	7.4010742	Reference			
Stroke						
No	9.5092	6.9696932	−5.621868	2.442903	−2.30	0.0219 *
Yes	25.1667	8.1083768	Reference			
Severity of disease						
Asymptomatic	5.5385	7.4129636	−3.295884	1.507726	−2.19	0.0294 *
Mild	5.5577	7.2660448	−3.364786	1.06918	−3.15	0.0018 **
Moderate	11.2455	7.2969359	−1.7349	1.191134	−1.46	0.1460
Severe	32.0000	7.2273743	Reference			

**Table 9:** Contingency table of outcome by age groups. *p* < 0.01 (**).

Outcome	18–29	30–39	40–49	50–59	60–69	70–79	80 and Above	Total	*p*-Value
Cured and discharged	85	99	80	55	60	28	12	419	0.0013 **
	100%	100%	98.77%	98.21%	95.24%	87.50%	85.71%		
Death	0	0	1	1	3	4	2	11	
	0%	0%	1.23%	1.79%	4.76%	12.50%	14.29%		
Total	85	99	81	56	63	32	14	430	

**Table 10:** Summary of main results and important highlights of current study in comparison to previous studies.

Parameters		Main Results of Current Study	Results of Previous Studies	New Contributions/Important Highlights of Current Study
Clinical characteristics	Fever	Most frequent symptoms	Similar results seen [30,31,32,33]	Statistically more commonly in older patients of age groups 60–69 years, 70–79 years, and 80 years old and more compared to younger groups.
	Cough			
	Dyspnea			
	Ageusia	Other symptoms found	Nonspecific symptoms	Statistically higher in younger age groups than in older patients.
	Abdominal pain			
Co-morbidities	Diabetes	Most frequent co-morbidities	Diabetes and HTN were the most common co-morbid conditions [22,39]	a. More 60–69 years older patients had statistically higher HTN, DM, CHF, and hypothyroidism. b. Diabetes and HTN were common in ages 70–79 years, 50–59 years, and 40–49 years. In contrast, asthma and other respiratory disorders were comparatively higher in the younger age group of 30–39 years. c. More males suffered HTN and cardiovascular disease, while obesity, hypothyroidism, and vitamin D deficiencies were comparatively higher in females.
	Hypertension			
	Asthma/respiratory disorders			
Predictors of severity	Pneumonia on chest X-ray	Statistically significant differences found for pneumonia, CVD, stroke, ICU, and mechanical ventilation	Older age, male sex, and presence of co-morbidities associated with severe disease at admission [22]	a. The age groups of 70–79 years and 60–69 years required intubation, which was statistically significant compared to younger patients. b. A higher percentage of males were given oxygen support on arrival and treated with systemic steroids. c. Age groups 70–79 years and 80 and above were significantly associated with in-hospital mortality. d. Nearly 55.67% of “70–79 years” and the majority of “80 and above” were suffering severe disease at the time of admission, and nearly 15% of each group succumbed to the disease.
	Co-morbid conditions such as CVD, stroke			
	ICU stay			
	Mechanical ventilation			
Length of hospital stay		The median period of hospital stay was six days	Similar results seen [49]	It was significantly longer in patients with severe disease who needed oxygen support or mechanical ventilation, as expected, and also longer in patients with CVD or stroke and administered systemic intravenous steroids.

## Data Availability

Data that underlie the results reported in this article, after deidentification, protocol, and statistical analysis will be available on request. Researchers should provide a methodologically sound proposal.
